# Supplementary material for: Freezing of few nanometers water droplets
Source: Nat Commun. 2021 Nov 30;12:6973. doi: 10.1038/s41467-021-27346-w (PMC8632967; doi:10.1038/s41467-021-27346-w)
Supplement: Supplementary file 1 — Supplementary information [file 41467_2021_27346_MOESM1_ESM.pdf]

Supplementary Information for

Freezing of few nanometers water droplets

Alireza Hakimian[1], Mohammadjavad Mohebinia[2], Masoumeh Nazari[1], Ali Davoodabadi[1], Sina Nazifi[1], Zixu Huang[1], Jiming Bao[2], and Hadi Ghasemi[1,3*]

[1]Department of Mechanical Engineering, University of Houston, 4726 Calhoun Rd, Houston, Texas 77204, USA

[2]Department of Electrical and Computer Engineering, University of Houston, 4726 Calhoun Rd, Houston, Texas 77204, USA

[3]Department of Chemical and Biomolecular Engineering, University of Houston, 4726 Calhoun Rd, Houston, Texas 77204, USA

*Correspondence to: hghasemi@uh.edu.

Supplementary Figures

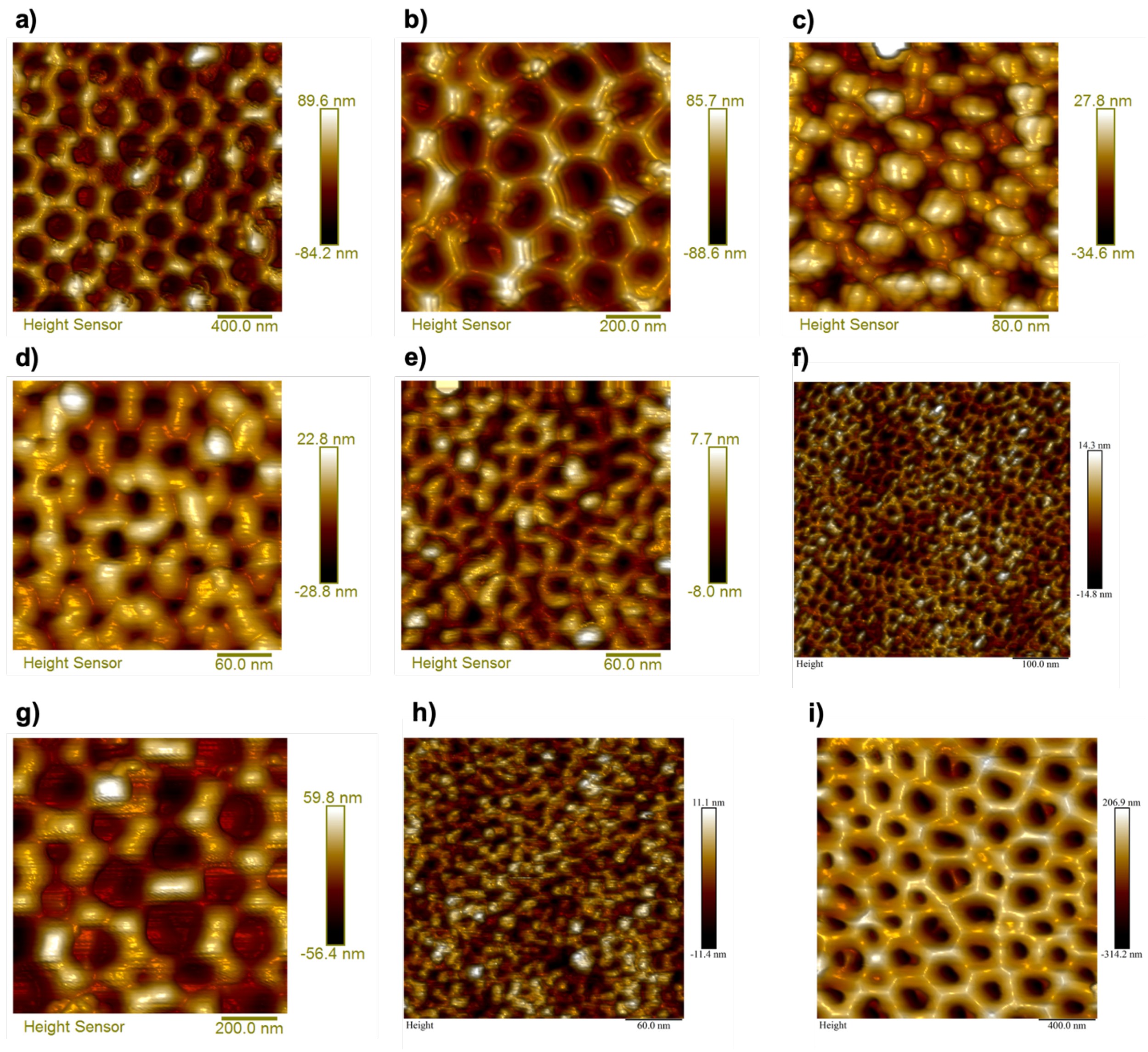

Supplementary Figure 1. Surface morphology of AAO membranes. Pore geometry of membranes with pore diameter of (a) 150 nm (b) 80 nm (c) 40 nm (d) 20 nm (e) 10 nm (f) 5 nm (active layer) (g) 5nm (support layer) (h) 2 nm (active layer) (i) 2 nm (support layer).

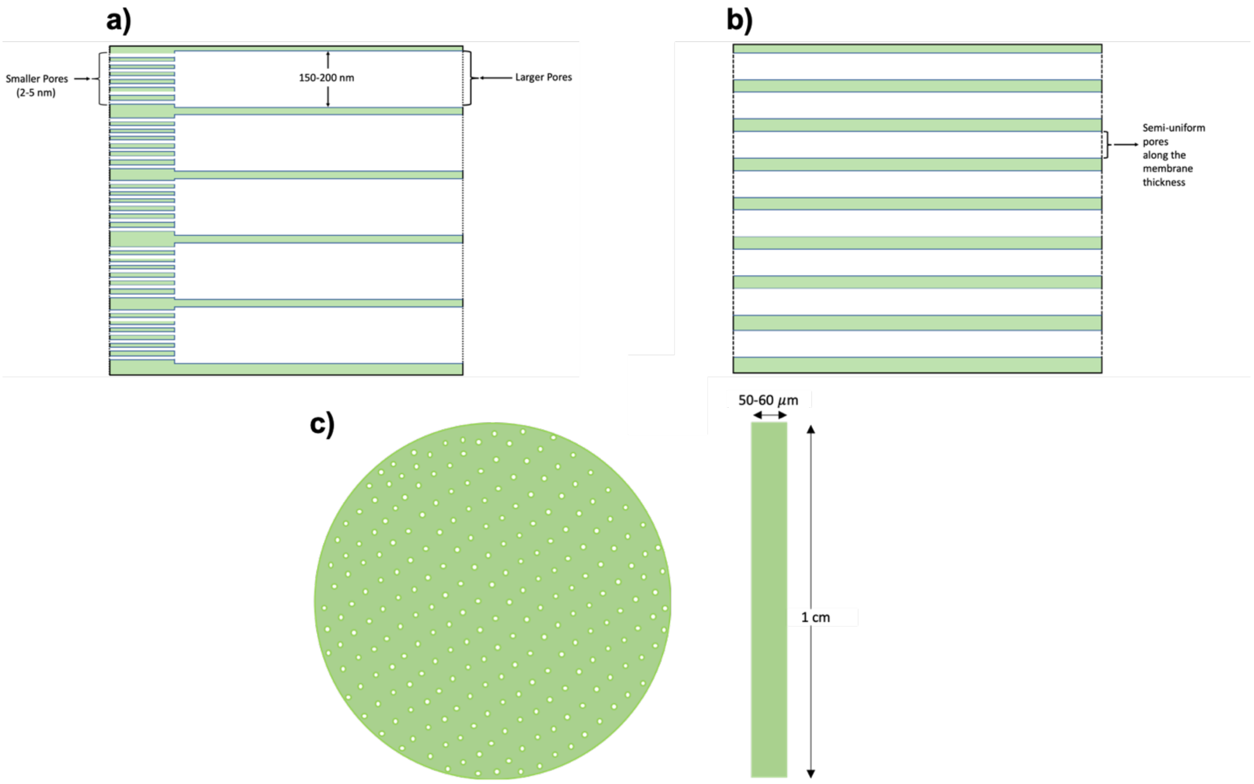

Supplementary Figure 2. Schematic of AAO membranes used in this research. These membranes are either (a) Anisotropic or (b) Isotropic. 2 and 5 nm AAO membranes are anisotropic and the rest of the membranes are isotropic. Isotropic membranes have uniform pores and constant pore diameter across membrane thickness. Anisotropic membranes consist of two layers: the first layer is an active layer with a pore diameter of 2 or 5 nm. The second layer is the supporting layer with a pore diameter of 150-200 nm which acts as a support for the active layer. The support layer forms most of the membrane thickness. Note that 2 nm and 5 nm membranes are only available in the form of anisotropic. (c) Schematic of top-down and side view of AAO membranes.

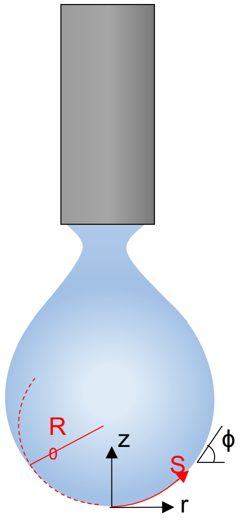

Supplementary Figure 3. Schematic of pendant drop experiments.

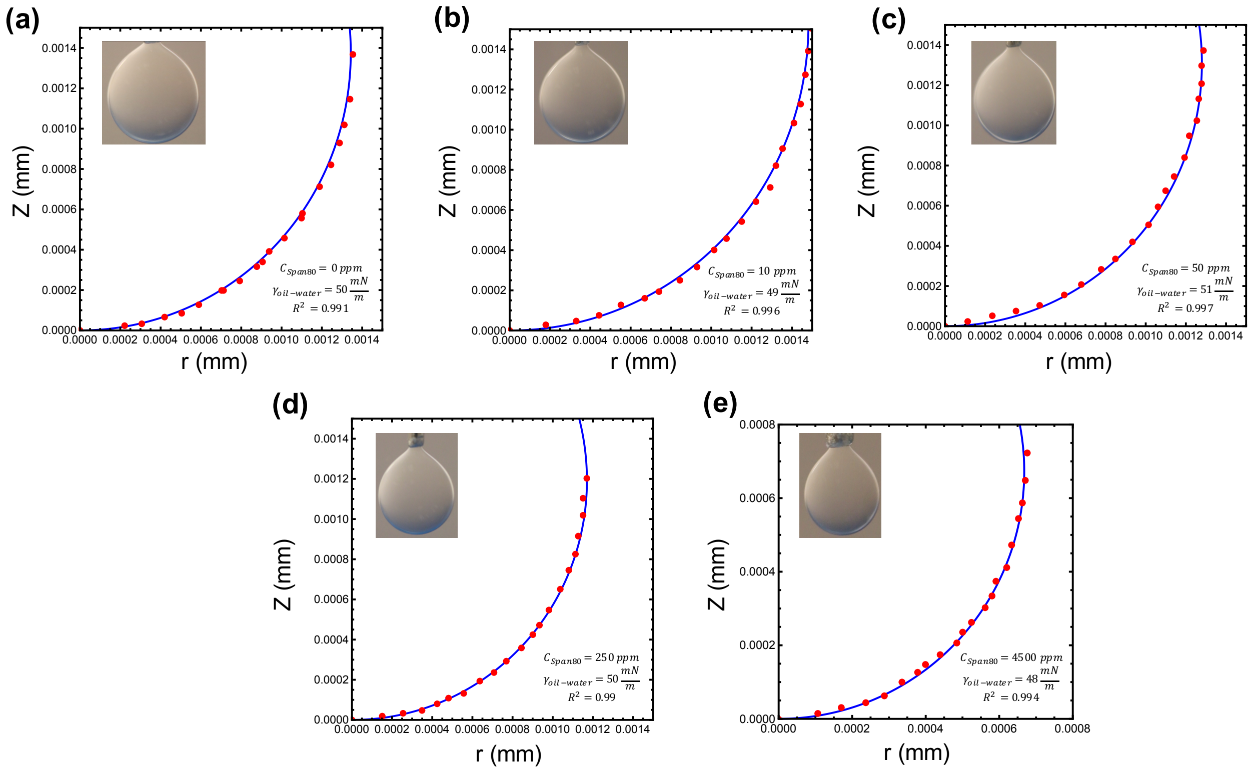

Supplementary Figure 4. The measured shape of water droplets suspended in octane are compared with the calculated shape to determine the surface tension of octane-water interface as a function of Span80 concentration. (a) 0 ppm Span80 concentration, (b) 10 ppm Span80 concentration, (c) 50 ppm Span80 concentration, (d) 250 ppm Span80 concentration, and (e) 4500 ppm Span80 concentration. Note that even up to the concentration of 4500 ppm, the changes in surface tension is less than 5%.

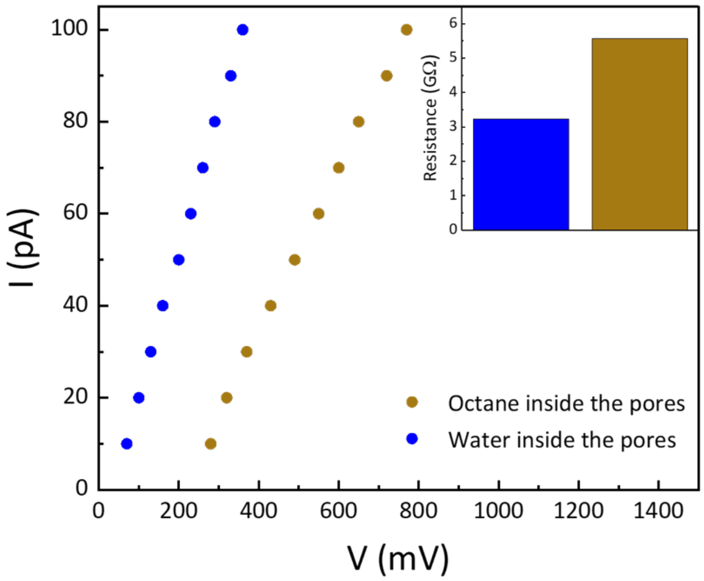

Supplementary Figure 5. Comparison between I-V curves obtained for the cases of pores of filled octane and the cases of pores with water nanodroplets inside. These experiments are conducted on the same experimental setup and at the same ambient temperature (23 °C).

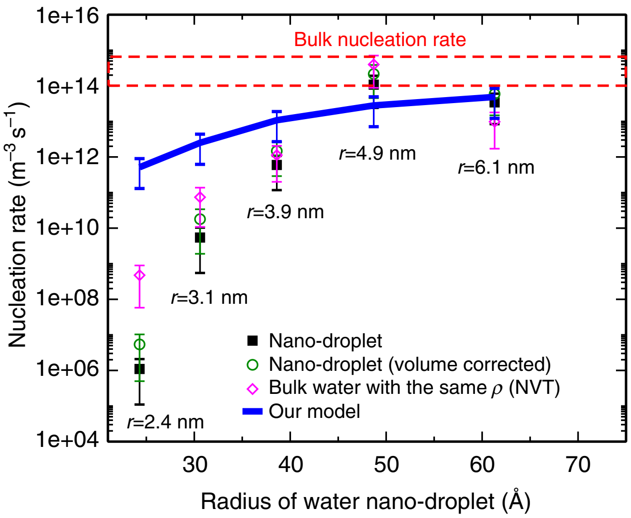

Supplementary Figure 6. Size dependence of ice nucleation rates in the mW water droplets at 230 K[4].The error bar denotes variations in the nucleation rate.

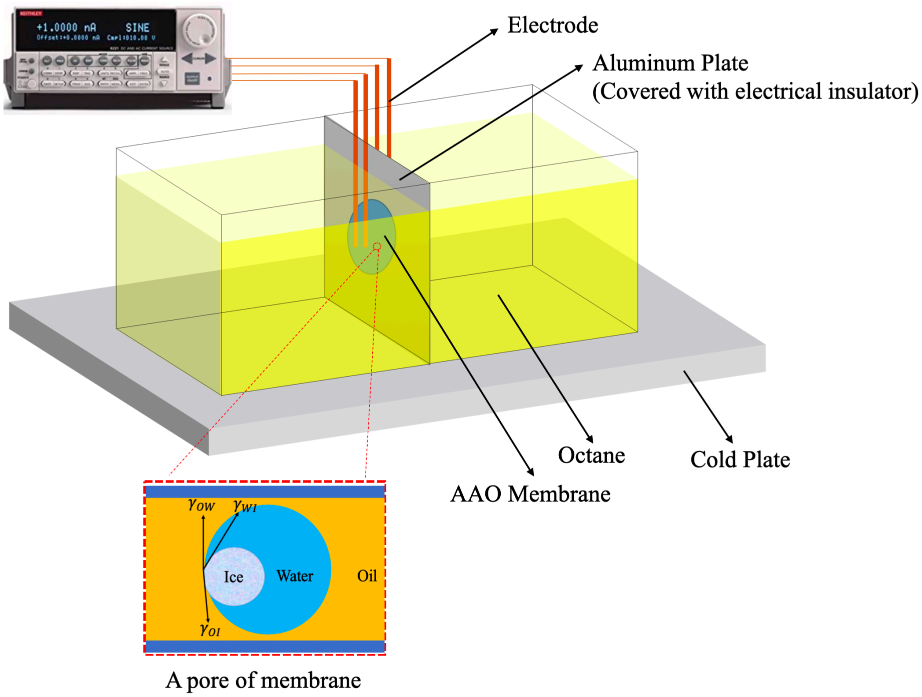

Supplementary Figure 7. The schematic of the experimental setup for electrical resistance metrology. The I-V curve across the pores is measured at a given temperature to probe the onset of water-ice phase change, T_N.

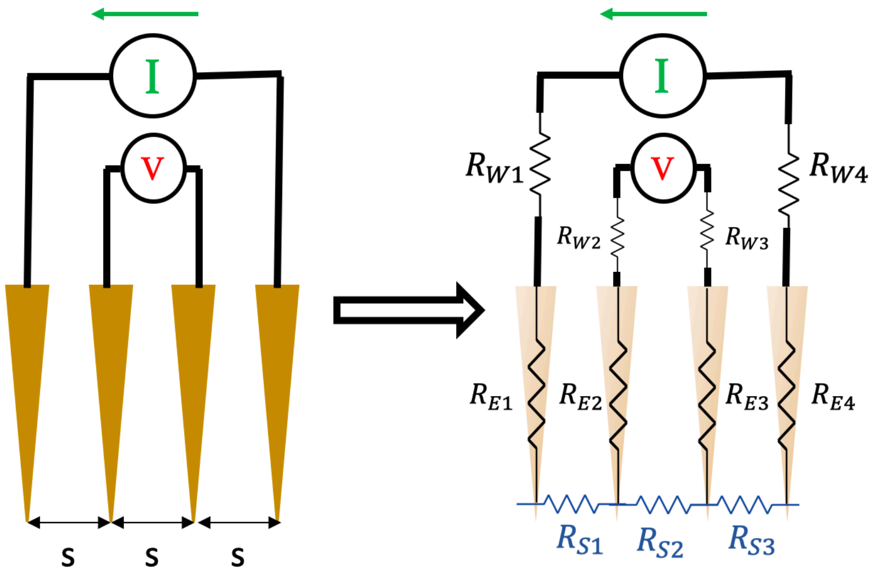

Supplementary Figure 8. 4-point probing method concept

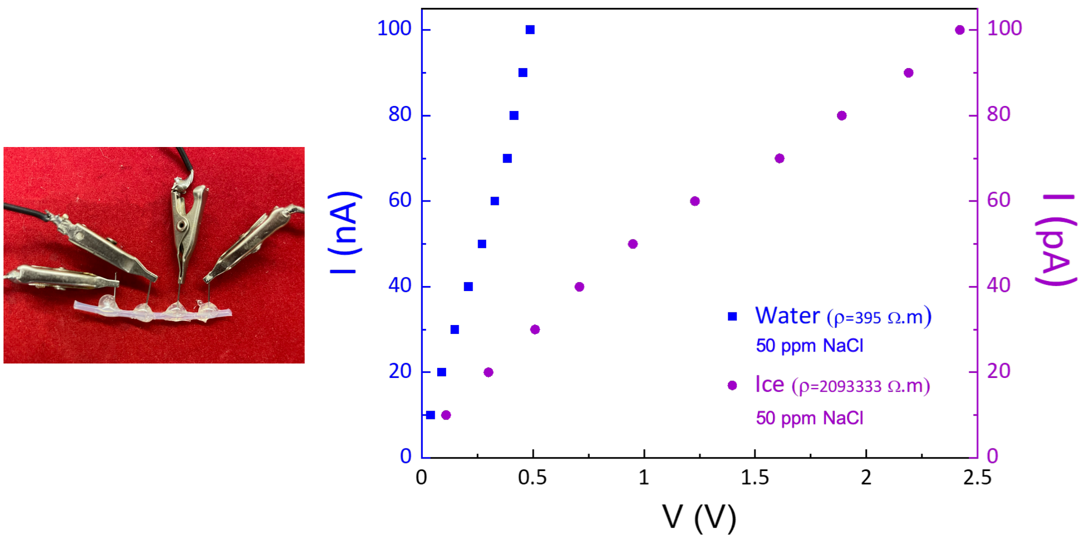

Supplementary Figure 9. The specific electrical resistivity of water solution and corresponding ice phase were measured in a plastic tube with inner diameter of 1 mm.

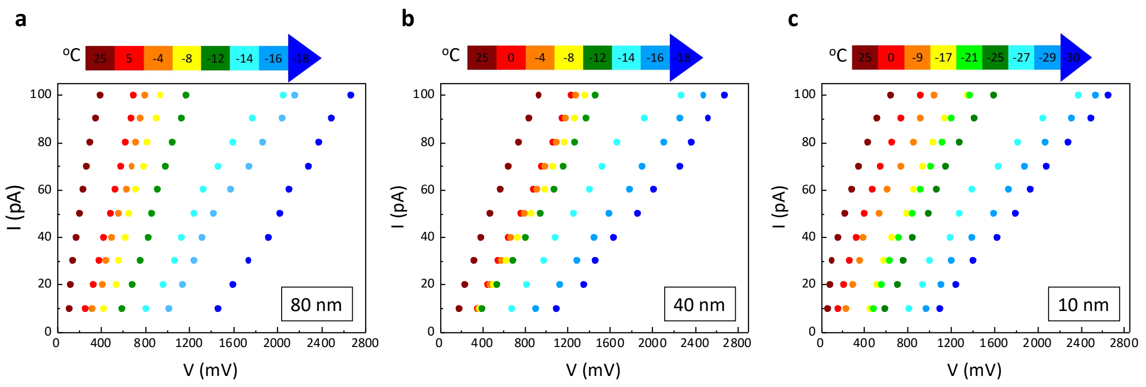

Supplementary Figure 10. I-V curve across nanopores measured as a function of temperature for water nanodroplets in the pores of membranes in the sizes of (a) 80 nm (b) 40 nm and (c) 10 nm. The average of two temperatures between which the jump in resistance is observed is assigned as ice nucleation temperature (T_N).

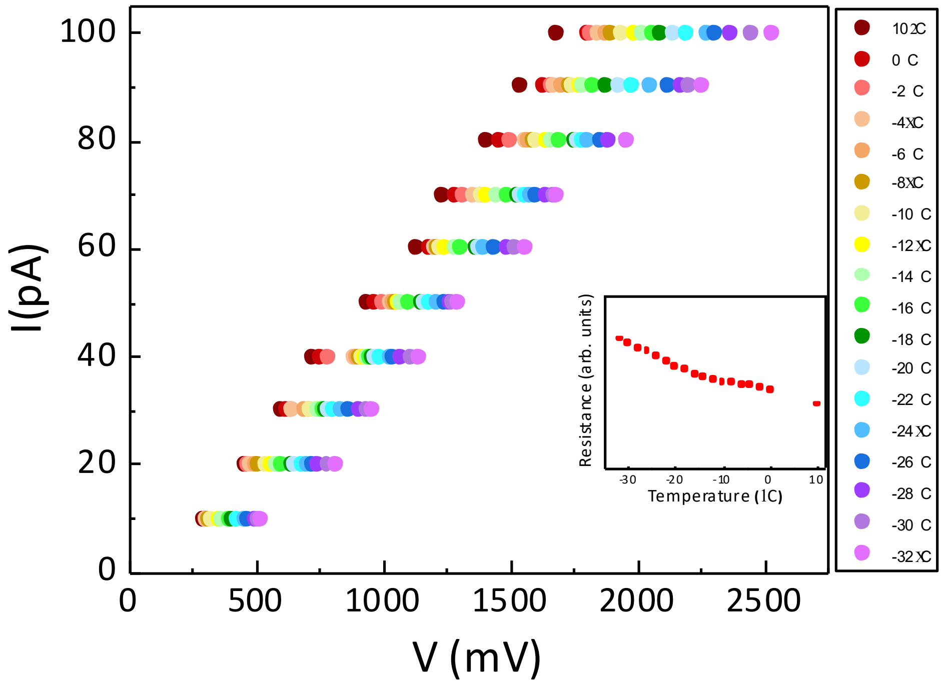

Supplementary Figure 11. I-V curves measured across the membrane when there is no water inside the pores in the membrane with pore diameter of 80 nm. There is no nonlinear jump in electrical resistance down to -32°C.

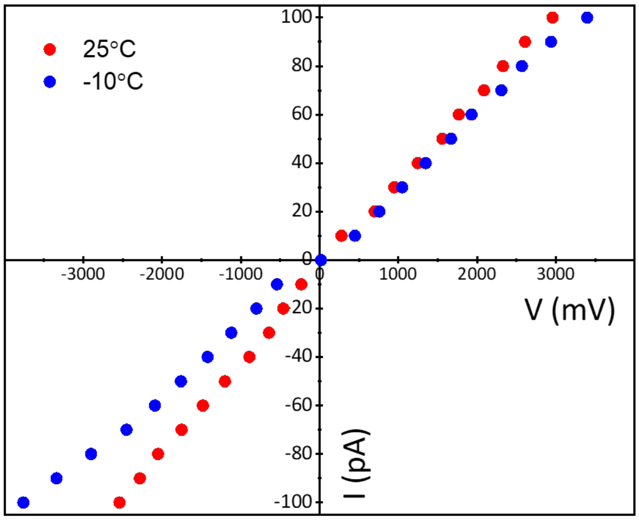

Supplementary Figure 12: The complete I/V curve for a membrane with pore diameter of 80 nm filled with only Octane at two temperatures which indicates Ohmic characteristic of this system.

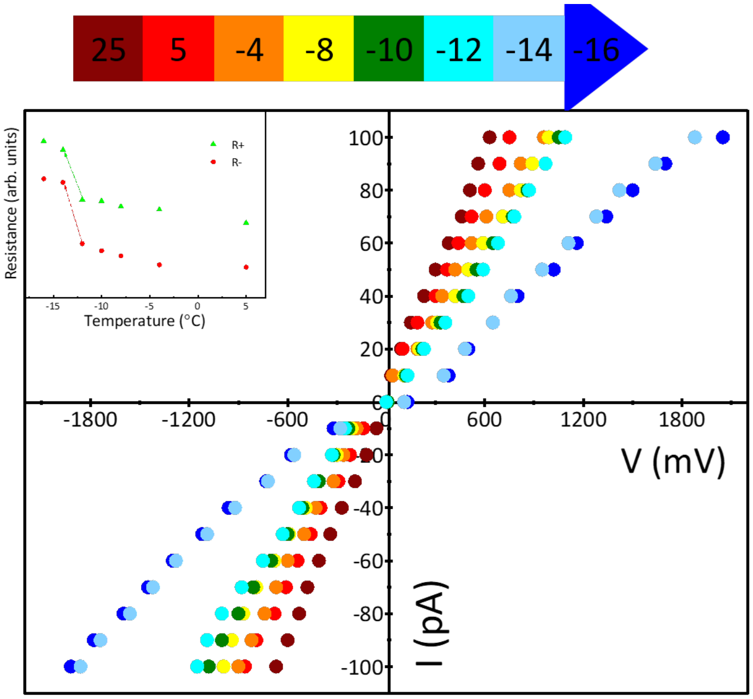

Supplementary Figure 13: The complete I/V curve for a membrane with pore diameter of 80 nm filled with water surrounded by Octane liquid. The temperature of the system is gradually decreased to probe water-ice transformation temperature. The nonlinearity in the resistance indicates phase transformation temperature. The system shows Ohmic characteristics.

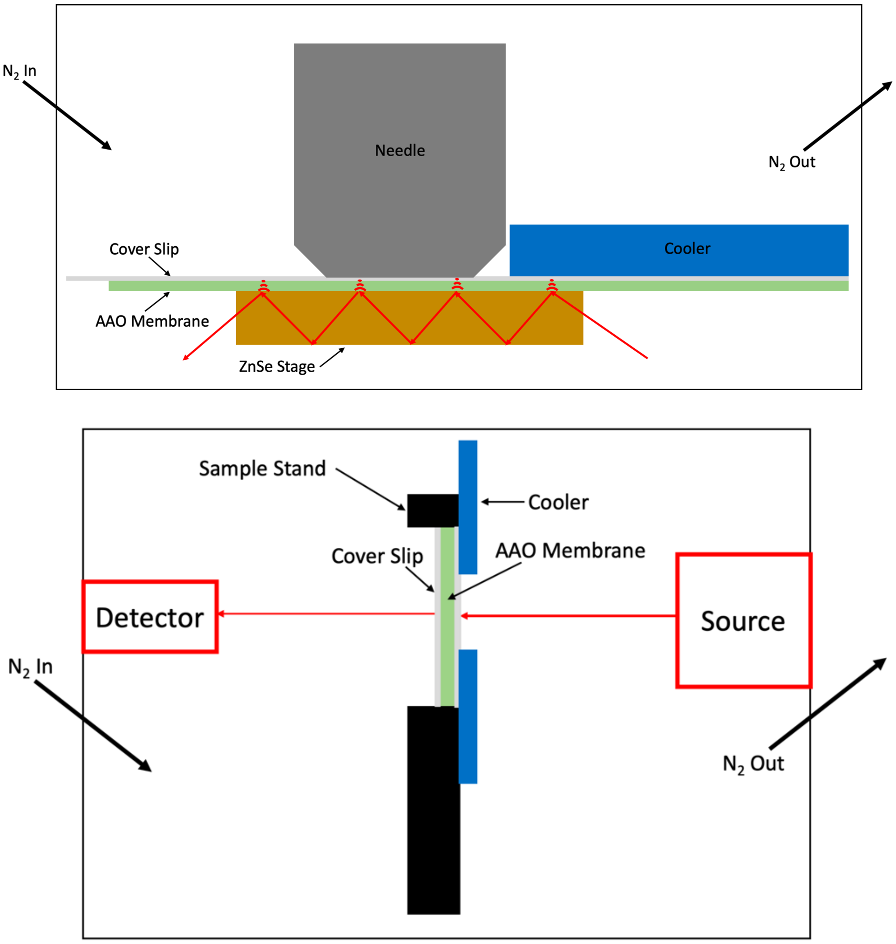

Supplementary Figure 14. Schematic of FTIR analysis setup that is used.

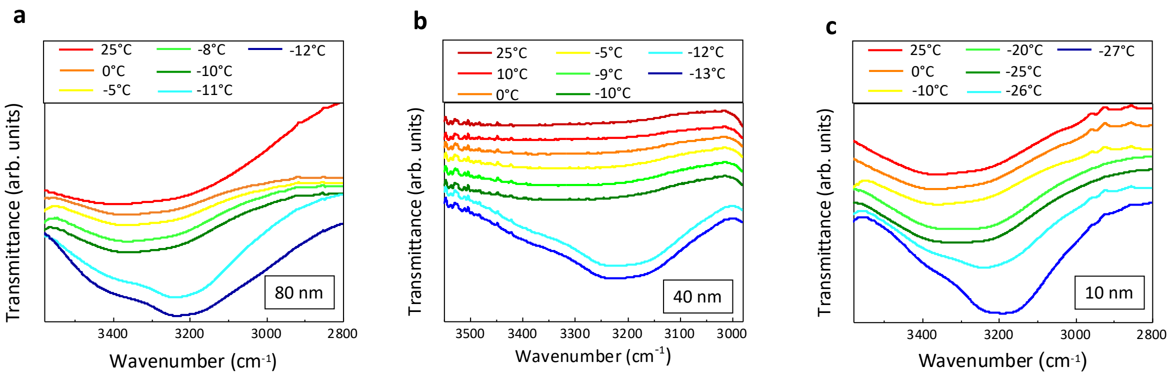

Supplementary Figure 15. FTIR metrology of nano-confined water droplets in the pores of the membrane with diameters of (a) 80 nm (b) 40 nm and (c) 10 nm encapsulated by octane. The temperature of the system is reduced in a quasi-static approach.

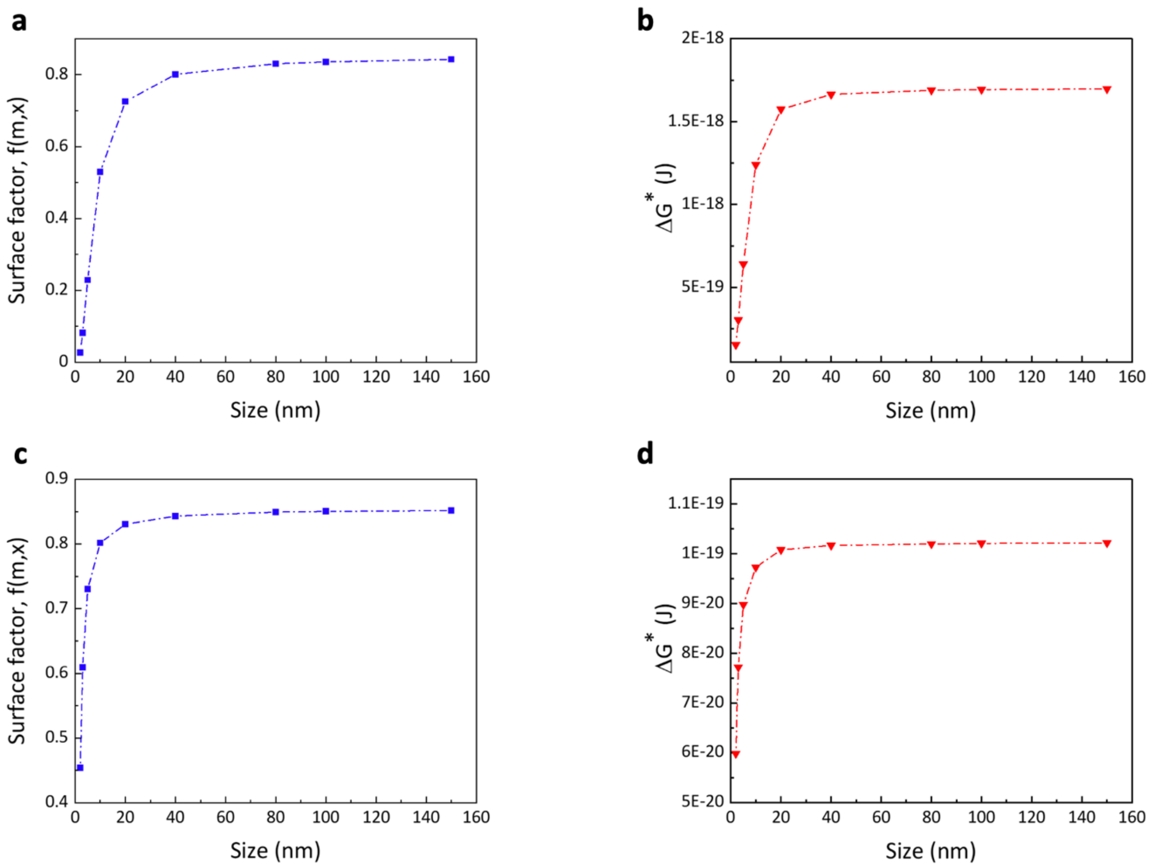

Supplementary Figure 16. (a) The role of interface geometry (x) on surface function at T= -10℃ and (b) Gibbs energy barrier (ΔG^*) is shown as a function of length scale by considering the effect of interface geometry at T=-10℃. (c) The role of interface geometry (x) on surface function at T= -41℃ and (d) changes in ΔG^* is shown as a function of length scale by considering the effect of interface geometry at T=-41℃.

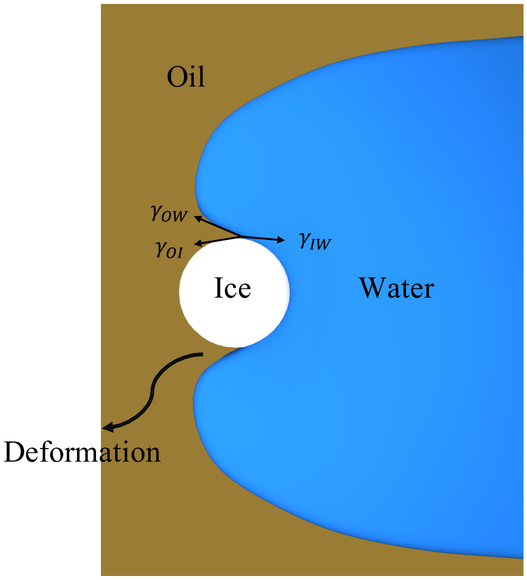

Supplementary Figure 17. Deformation of oil-water interface as ice nucleates at the interface. The deformation is caused by unbalanced $\gamma_{IW}\sin\theta$ force leading to local convex and concave coordinate (ripples) at the oil-water interface. This deformation distinguishes ice nucleation on the soft matter from those of stiff-solid interface.

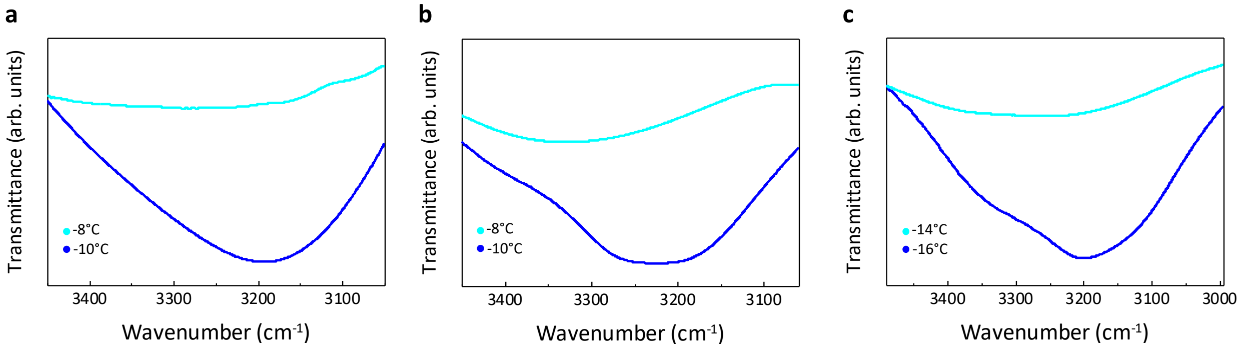

Supplementary Figure 18. FTIR spectrum of nanodroplets in membrane with pore diameter of 10 nm surrounded by PDMS/Octane with different moduli (different ratio of prepolymer to curing agent) (a) 10:1 (b) 30:1 and (c) 60:1.

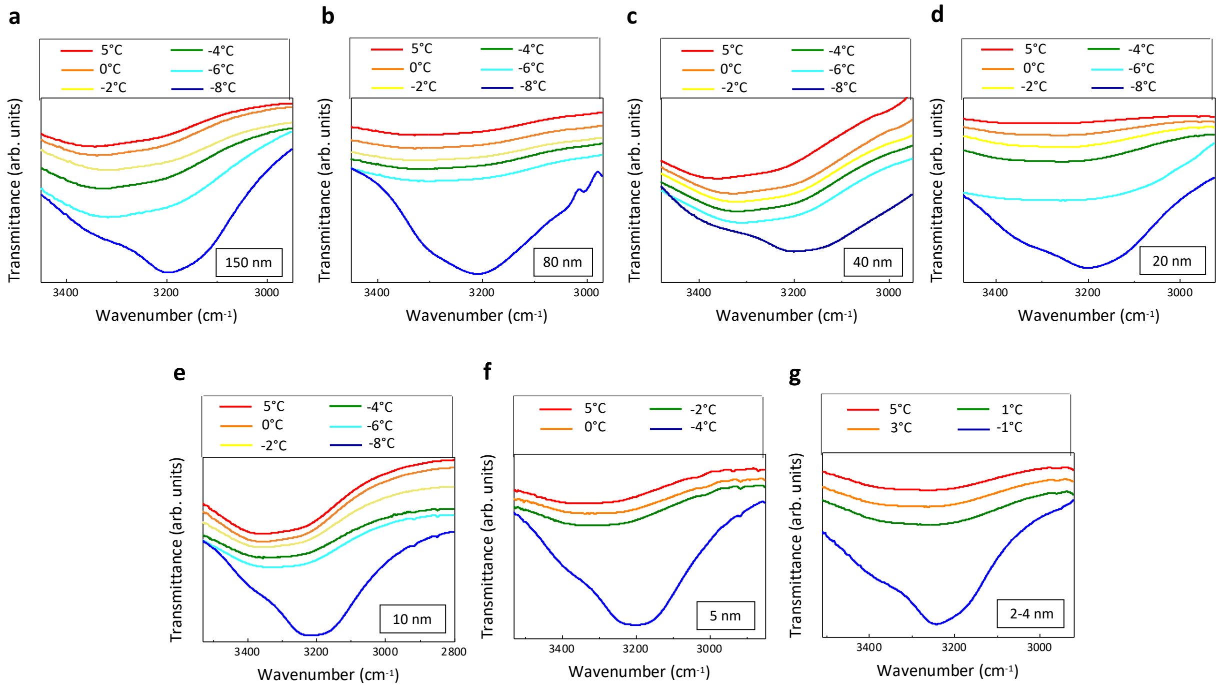

Supplementary Figure 19. FTIR spectrum of nanodroplets in various pore dimensions when pores are filled with water without addition of oil around them. (a) For 150 nm nanodroplets, at the temperature of 7°C, the OH stretch peak is narrowed and red-shifted to wavenumber of ~3200 cm^{-1}. This red shift indicates the water-ice phase change and provides T_N value at this length scale. (b) The red shift occurs at temperature of -7°C for 80 nm water droplets as well. (c) The red shift occurs at temperature of -7°C for 40 nm water droplets as well. (d) For 20 nm nanodroplets, at the temperature of -7°C, the OH stretch peak is narrowed and red-shifted to wavenumber of ~3200 cm^{-1}. This red shift indicates the water-ice phase change and provides T_N value at this length scale. (e) The red shift occurs at temperature of -7°C for 10 nm water droplets as well. (f) For 5 nm water droplets, the narrowing of OH stretch peak occurs at the temperature of -3°C. (g) The T_N value for 2 nm droplets becomes ~ 0°C.

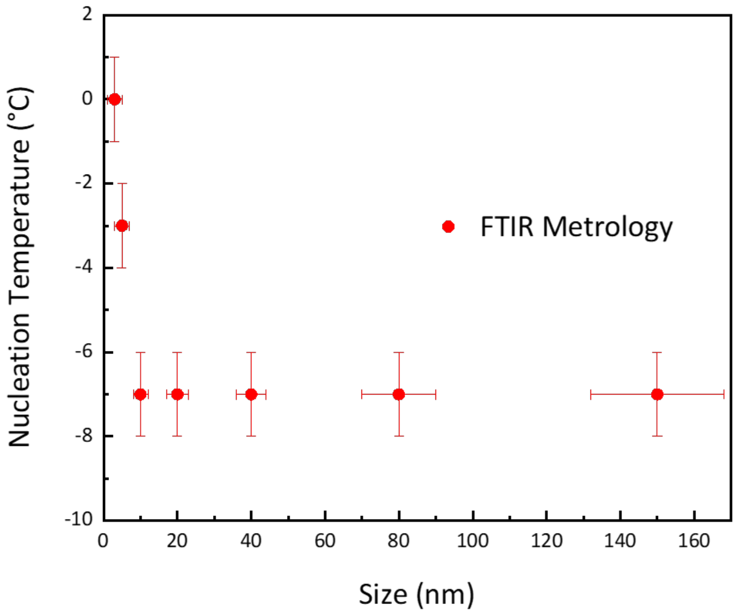

Supplementary Figure 20. The nucleation temperature of water droplets, when the membrane is filled with water without addition of oil, measured through FTIR metrology is shown as a function of length scale. The horizontal error bars denote variations in the dimension of the water droplets, and the vertical errors bar denotes experimental errors in the measurement of nucleation temperature.

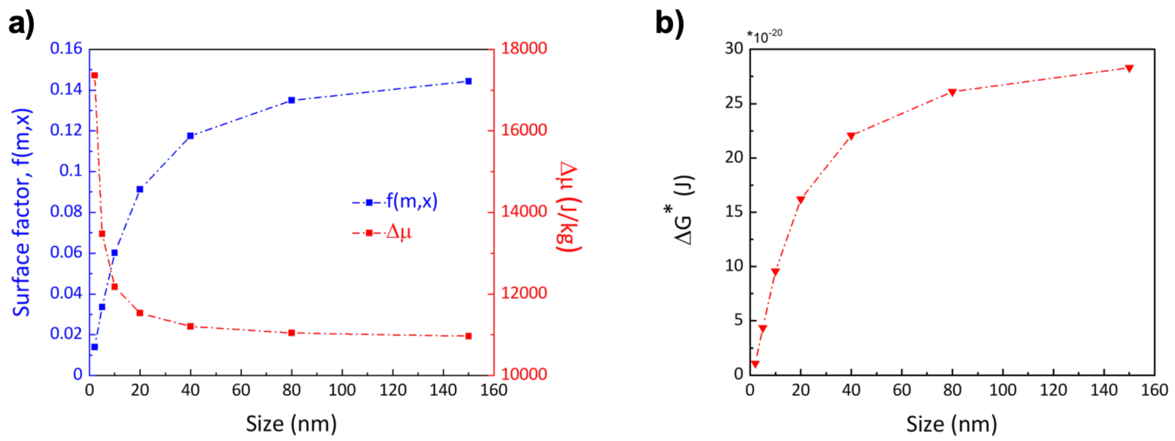

Supplementary Figure 21. (a) The role of interfacial curvature on surface function and dependence of chemical potential difference on length scale is shown when pores are filled with water without addition of the oil. (b) In this scenario, the Gibbs energy barrier for water-ice phase change of nanodroplets is shown as a function of the diameter of nanodroplets.

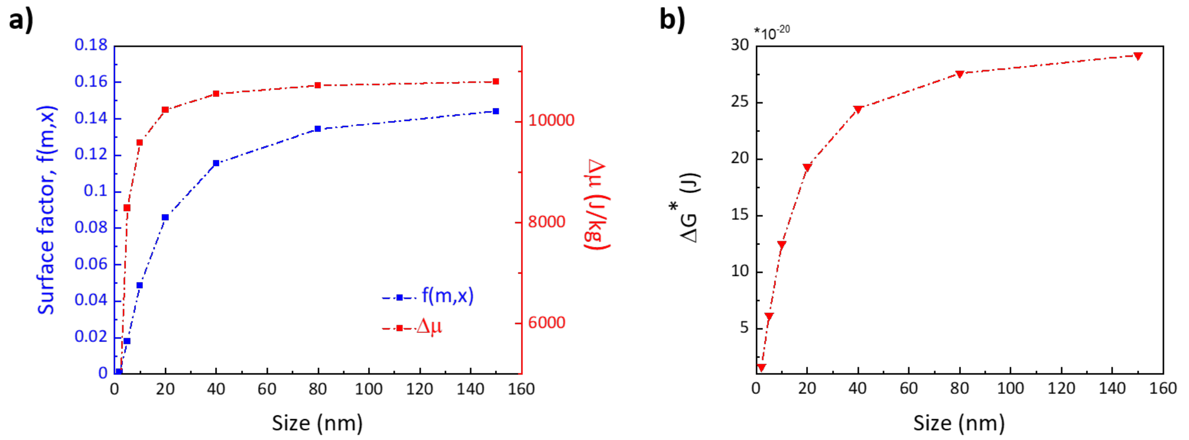

Supplementary Figure 22. (a) The role of interfacial curvature on surface function and dependence of chemical potential difference on length scale is shown when pores are filled with water and considering the assumption that added oil just has contact with water at the ends and does not wet the pore wall. Thus, water is in contact with pore walls along its length. (b) In this scenario, the Gibbs energy barrier for water-ice phase change of nanodroplets is shown as a function of diameter of nanodroplets.

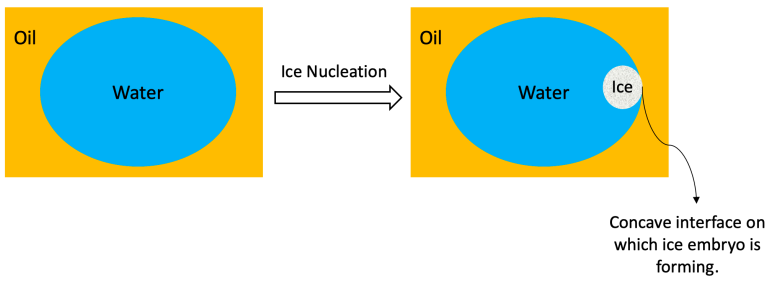

Supplementary Figure 23. Schematic of water nano droplet encapsulated in oil and the formation of ice embryo inside water.

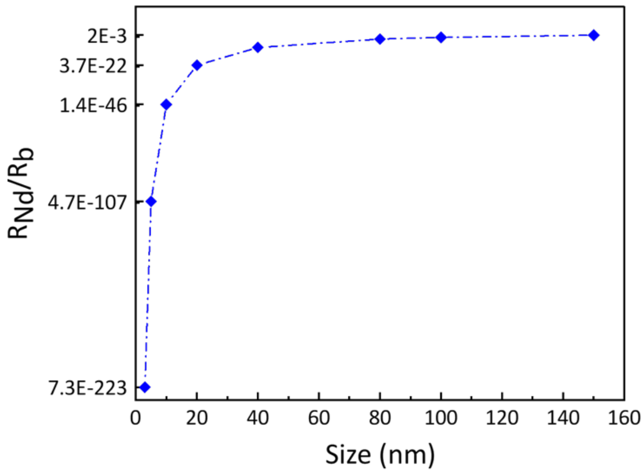

Supplementary Figure 24. The normalized ice nucleation rate of nanodroplets with respect to bulk water is shown as a function of length scale at T=-10℃.

Supplementary Tables

Supplementary Table 1: Induced pressures for confined nanodroplets for different potential surrounding fluid

Size(nm)	P (bar)					
	Dichloromethane	Carbon tetrachloride	Hexane	Heptane	Octane	Decane
100	5.77	9.18	10.40	10.58	10.71	9.18
80	7.18	11.43	12.95	13.18	13.33	11.43
50	11.43	18.18	20.60	20.96	21.21	18.18
20	28.41	45.18	51.20	52.10	52.71	45.18
10	56.71	90.18	102.20	104.00	105.21	90.18
5	113.31	180.18	204.20	207.80	210.21	180.18
2	283.11	450.18	510.20	519.20	525.21	450.18

Supplementary Table 2: Surface tension of oil-water interface as a function of Span80 concentration

Concentration of Span 80	γ_{ow} in literature (mNm^{-1})	Measured γ_{ow} (mNm^{-1})
0 ppm	51	50
10 ppm		49
50 pm		51
250 ppm		50
4500 ppm		48

Supplementary Table 3: Volume of liquid in the pores measured by QCM metrology

Pore Diameter (nm)	Pore Density ($\frac{1}{cm^2}$)	Thickness of membrane (μm)	Theoretical weight of water inside the pores ($\frac{\mu g}{cm^2}$)	Fs (MHz)		Weight of water inside the pores ($\frac{\mu g}{cm^2}$)
				**Empty membrane**	**Filled membrane**	
20	5×10^{10}	50	785	10.0036792	10.00349456	816.99
10	1.6×10^{11}	50	628	10.0036887	10.00357321	511.02
Active layer: 5<br>Support layer: 150	Active layer: 9×10^{11}<br>Support layer: 1.5×10^{9}	Active layer: 3<br>Support layer: 60	1616.12	10.0036916	10.00339504	1312.21

Supplementary Table 4: Volume of water inside each single pore of membranes with different pore diameter

**Pore Diameter (nm)**	**Volume of water inside each pore (pL)**
150	$8.83*10^{-4}$
80	$2.51*10^{-4}$
40	$6.28*10^{-5}$
20	$1.57*10^{-5}$
10	$3.92*10^{-6}$
5	$1.17*10^{-7}$
2-4	$4.24*10^{-8}$

Supplementary Table 5: Critical nucleolus radius as a function of temperature

**Temperature (℃)**	**r_c (nm)**
-10	4.21
-15	2.92
-20	2.23
-25	1.80
-30	1.50
-35	1.29
-40	1.13
-45	1.00
-50	0.89

Supplementary Table 6. Nucleation temperature of water nano-droplet confined in 10 nm nanopores and encapsulated with PDMS/Octane with different moduli[11].

**Base: Crosslinker (weight ratio)**	**Young modulus (kPa)**	**TN (°C)**
10: 1	1300	-9
30: 1	120	-9
60: 1	3	-15
Just Octane	0	-26

Supplementary Notes

Supplementary Note 1. Surface topography of pores

The geometry and dimension of pores in the nanomembranes are probed with scanning probe microscopy (SPM) as shown in **Supplementary Figure 1**. The 5 and 2 nm membranes are made of two layers, the active layer with a small pore dimension and the supporting layer with pore dimensions of 150-200 nm. For 2 nm membranes, the pores have a size distribution of 2-4 nm. Furthermore, the cross-section of these nanomembranes is shown schematically in **Supplementary Figure 2**.

Supplementary Note 2. Selection of surrounding fluid of the nanodroplets

The included pressure inside the nanodroplets (P_{Nd}) is determined through the Laplace equation written as, $P_{Nd} - P_{atm} = \gamma_{ow}\,(C_1 + C_2)$, where P_{atm} denotes the atmospheric pressure equal to oil pressure, γ_{ow} is the interfacial tension of the oil-water interface, and C_1 and C_2 are the principal curvatures of the water-oil interface[1], **Supplementary Table 1**. Based on the volume of water in the pores, we expect the water nanodroplets are formed in the shape of elongated droplets in the pores. As the thickness of membranes is much larger than the diameter of pores (i.e. 50-60 μm thickness compared to the 2-150 nm pore diameter), the value of C_2 becomes negligible compared to C_1. Here, we considered that the value of C_1 is approximately inverse of the radius of pores.

Regarding the effect of Span 80 on interfacial tension, it should be noted that HLB (Hydrophilic–lipophilic balance) value for Span 80 is 4.3 which means that Span 80 is oil soluble and water-insoluble[2]. Furthermore, we used pendant drop method[3] to access the role of Span 80 concentration on octane-water interfacial tension. In these experiments, a water droplet is introduced in an octane medium with different concentrations of Span80 as shown below. The Bond number (Bo) is defined as

$$Bo = \frac{\Delta\rho\, g\, R_0^2}{\gamma_{ow}} \qquad (S1)$$

Where $\Delta\rho$ denotes the density difference between water and octane, g is the gravitational acceleration and R_0 is the radius of curvature at the drop apex, **Supplementary Figure 3**. The differential form of Young-Laplace equation in terms of arc length (s) is written as

$$\frac{d\phi}{d\bar{s}} = 2 - \;Bo\,\bar{z} - \frac{\sin\phi}{\bar{r}} \qquad (S2)$$

$$\frac{d\bar{r}}{d\bar{s}} = \;\cos\phi \qquad (S3)$$

$$\frac{d\bar{z}}{d\bar{s}} = \;\sin\phi \qquad (S4)$$

where the bar indicates dimensionless values scaled by R_0. The boundary conditions are

$$@\, s = 0:\ \bar{r} = 0;\ \bar{z} = 0\ ;\ \phi = 0$$

The input to this system of equations is Bo or specifically γ_{ow}.

Once this value is given, one can solve this system of equations numerically. We determined numerically the shape of the droplet for a range of surface tension values and compared the calculated droplet shape with the one measured, **Supplementary Figure 4**. For each considered value of surface tension, we determined the coefficient of determination (R^2) between the calculated shape and measured shape. The surface tension with the highest value of R^2 provides gives us γ_{ow} through pendant droplet method, **Supplementary Table 2**. As a control experiment, we compared the measured interfacial tension for octane-water with the reported value in the literature which shows less than 5% error.

Supplementary Note 3. The existence of water nanodroplets in the pores and volume effect

The electrical conductance across nanopores of 80 nm is measured through the four-probe method. If the pores are fully filled with octane, the electrical resistance is approximately 5.5 GΩ, while the introduction of nanodroplets in these pores drops the resistance to approximately 3 GΩ. Note that the existence of any blocked air bubble in the pore would result in the insulating characteristics of these pores.

We performed quartz crystal microbalance (QCM) measurement on the membranes before and after filling with water to determine the volume and length of water nanodroplets inside the pores. A piece of membrane was mounted on a gold chip before and after filling with water. This chip was placed in an electrochemical quartz crystal microbalance (Gamry's eQCM 10M). The weight of water inside the pores was measured based on the changes in the resonant frequency of the oscillating quartz crystal before and after filling with water. We also compared the measurements with the theoretical values calculated from the membrane properties provided by the vendor. The results are tabulated in **Supplementary Table 3.** The volume of water within the pores is 0.817 $\mu L\ cm^{-2}$ for 20 nm pores, 0.511 $\mu L\ cm^{-2}$ for 10 nm pores, and 1.312 $\mu L\ cm^{-2}$ for 5 nm pores.

To clarify the droplet volume effect, we should note that the droplets in these pores are elongated ellipsoid droplets with a smaller diameter in the range of few nm while the other diameter in tens of micrometer. The approximate volume of isolated water droplet in the pores are given in **Supplementary Table 4.**

We compared the volume of these droplets with the volume of water droplets reported by Li *et al.*[4], **Supplementary Figure 6**. As shown, for water droplets higher than 6.1 nm, the ice nucleation rate is similar to bulk water. The volume of 6.1 nm droplet is $9.5 * 10^{-10}$ pL. All of the droplets studied in this work have volumes at least 2 orders of magnitude higher than this limit. Thus, the volume effect on ice nucleation rate is insignificant in this study.

Supplementary Note 4. The experimental setup for E-resistance metrology

The schematic of the experimental setup is shown in **Supplementary Figure 7**. In this metrology, the nanomembrane was placed between two reservoirs separated by a heat conducting wall. We measured the temperature of two reservoirs during the experiments to ensure isothermal

conditions. The coordinates of the electrodes are fixed. Once the water nanodroplets are formed inside the pores, the temperature of the system in a quasi-static manner is reduced and the I-V curves across the pores are probed.

A 4-point probe consists of four electrical probes in a line, with equal spacing between each of the probes. Based on this method, a high impedance current source is used to supply current through the outer two probes; a voltmeter measures the voltage across the inner two probes (See **Supplementary Figure 8**). Due to the high impedance of voltmeter, no current flows through inner electrodes. In this case, the voltage drop is measured between two inner electrodes where wire resistances (R_{w2} and R_{w3}) and contact resistances (R_{c2} and R_{c3}) do not contribute in voltage measurement and it is just the resistance of sample (R_{s2}) which leads to the voltage drop (ΔV).

We explored one polarity where we applied current and measured the voltage. In fact, what is important here are non-linearities in the resistance and by this method, we can observe those non-linearities and jump in the resistance due to solid-liquid phase change.

Based on the 4-point probing method, the voltage is measured between two inner electrodes and these two electrodes are pretty close to the membrane, almost attached to the membrane. As a result, almost all of the voltage drop here comes from the membrane and the liquid inside the membrane and the changes observed in the resistance are due to the characteristic changes in the liquid inside the membrane. The liquid phase contains water and octane. Octane conductivity is due to the existence of a tiny amount of Span80 and conductivity of water is due to the sodium and chloride ions. Addition of sodium and chloride ions increases the conductivity of water due to ion conductivity. The most important feature is that, as water freezes, ions cannot move, and this increases ice resistance compared to water by more than three orders of magnitude. On the other hand, when there is no ion inside the water, the resistance of ice and water is in the same order of magnitude. It should be noted that these ions are not soluble in octane, as octane is completely non-polar, and after adding NaCl to octane it precipitates and octane resistivity, which is in order of Giga ohm (GΩ), does not change. Thus, sodium and chloride ions cannot be responsible for the charge transport inside the octane. Also, HLB (Hydrophilic–lipophilic balance) value for span 80 is 4.3 which means that span 80 is oil soluble and water-insoluble[2].

We took two independent approaches to show that the electrical resistance of water and ice could differ by more than three orders of magnitude.

Approach 1: As mobility of ions in ice approaches zero, the resistance of ice is close to its pure ice value as reported as 10^7 ohm.m. (C. Jaccard, Mechanism of the electrical conductivity in ice, Annals of the New York Academy of Sciences, 125, 390-400, 1965[5]). The electrical resistivity of water with 50 ppm of salt is reported ~100 ohm.m (Steve Felber, Water Fundamentals Handbook, DRI-STEEM, 2017[6]). This suggests that there are five orders of magnitude difference between resistance of water solution and pure ice. Please note that the membrane is made of many pores surrounded by water/octane and calculation of total resistance of the system will have high uncertainty.

Approach 2: In the second approach, we measured the electrical resistance of water solution and ice filled in a plastic tube with inner diameter of 1 mm as shown in **Supplementary Figure 9**. The tube was filled with water solution with 50 ppm NaCl and four-probe electrodes were attached to the tube. The specific electrical resistivity of the water is measured at 1 ^{o}C as 395 ohm.m. The

temperature of the system is dropped to -10 ºC allowing to ice form in the tube. The specific electrical resistivity of ice was measured as 2093333 ohm.m. That is, the specific resistivity of ice is ~5000 times higher than that of water solution.

Supplementary Note 5. Electrical resistance metrology

The water-ice phase transformation for different size nanodroplets is shown in **Supplementary Figure 10**. The non-linear shift in resistance across the pores indicates the phase change temperature (T_N).

We measured temperature-dependent I-V curves for octane-only filled membranes for 80 nm membrane and results are shown in **Supplementary Figure 11**. From this figure, we cannot observe any non-linearity down to -32°C. However, we observed non-linearity in resistance in water-filled 80 nm membranes around -12°C. This proves that non-linearities in resistance are not due to Octane.

We conducted the complete I-V experiments for pore dimension of 80 nm in two cases: (1) Pure Octane case at two temperatures of -10 and 25 ºC and (2) for water droplets surrounded by the Octane. The results for case (1) are shown in **Supplementary Figure 12** suggesting Ohmic behavior. The results for case (2) are shown in **Supplementary Figure 13** that indicate the non-linearity in the system resistance is observed in both positive and negative domains with Ohmic characteristics. Note that the minimum current value with the source meter system is 10 pA and we have high uncertainty in low current region.

Supplementary Note 6. FTIR metrology

The FTIR experimental setups are shown below, **Supplementary Figure 14**. We used peltier coolers to control the sample temperature. In Attenuated Total Reflectance (ATR) mode, once the sample was placed on the FTIR stage, the peltier coolers were placed on a part of the membrane covered by a coverslip. For probing the sample temperature, a thermocouple was attached to the edge of the membrane. Because the membrane is placed between coolers, FTIR stage and coverslip, the vapor condensation from the surrounding environment is minimal. Furthermore, to prevent any possible frost formation, nitrogen gas was purged in the setup continuously. In the transmission mode, the membrane was sandwiched between coverslips and mounted on a stand and the light passed through the sample. C-H peaks from oil are subtracted using background and the resolution of the instrument is 4 cm^{-1}.

The water-ice phase transformation probed by FTIR metrology is shown in **Supplementary Figure 15**. The narrowing and shift in OH stretch peak indicate the phase change temperature (T_N).

Supplementary Note 7. Heterogeneous nucleation of nanodroplets

Gibbs energy barrier for heterogeneous ice nucleation is written as[7,8]:

$$\Delta G^* = \frac{16\pi\gamma_{IW}^3}{3(\rho\Delta\mu)^2} f(m,x) \qquad (S5)$$

where γ_{IW} denotes interfacial tension between water and ice, $\Delta\mu$ is the chemical potential difference between ice and water phases and $f(m,x)$ denotes the surface function. Surface function depends on interfacial tensions, m, and surface geometry, x, defined as follows

$$m = \frac{\gamma_{OW} - \gamma_{OI}}{\gamma_{IW}} = \mathrm{Cos}\,\theta \qquad (S6)$$

where γ_{OW} is oil-water interfacial tension, γ_{OI} is oil-ice interfacial tension, γ_{IW} is ice-water interfacial tension and θ is the ice embryo contact angle with the oil-ice interface. x depends on interfacial geometry and is written as

$$x = \frac{R}{r_c} \qquad (S7)$$

where r_c is the critical nucleolus radius and R is the radius of curvature of the oil-water interface. r_c is written as[7,9]

$$r_c = \frac{2\gamma_{IW}}{\Delta G_v} \qquad (S8)$$

$$\Delta G_v = \Delta H_v \frac{T_m - T}{T_m} \qquad (S9)$$

Where ΔG_v denotes the free energy difference per unit volume between water and ice, ΔH_v denotes enthalpy of phase change per unit volume, T_m is the melting temperature of ice, and T is the temperature of the system. Also, ice-water interfacial tension is estimated as[8]

$$\gamma_{IW} = 23.24\left(\frac{T}{235.8}\right)^{0.35} \qquad (S10)$$

The surface function, $f(m,x)$, for a concave surface is written as[8,10]

$$f(m,x) = \frac{1}{2}\left\{1 - \left(\frac{1+mx}{g_c}\right)^3 - x^3\left[2 - 3\left(\frac{x+m}{g_c}\right) + \left(\frac{x+m}{g_c}\right)^3\right] + 3mx^2\left(\frac{x+m}{g_c} - 1\right)\right\} \qquad (S11)$$

$$g_c = (1 + x^2 + 2mx)^{1/2} \qquad (S12)$$

If one considers the effect of oil-water interface geometry, x, upon decreasing pore diameter, we observe a decrease in $f(m,x)$ and as a result a reduction in ΔG^*, as shown in **Supplementary Figure 16** for two different temperatures.

Here, we have distinguished heterogeneous and homogeneous modes of nucleation through Gibbs energy barrier (ΔG^*) analysis. If the nucleation in these pores were homogenous, we should not

observe any effect of confinement size on the nucleation temperature. For example, for the case of pure water in the confinement, the nucleation temperature varies from -7 to 0 C, **Supplementary Figure 19**.

To further confirm this, we performed another set of experiments with membrane with pore dimension of 10 nm where we switched the encapsulating Octane phase with PDMS with various modulus as shown in **Supplementary Table 6**. In these experiments, we initially filled the membranes with water. Mixtures of Sylgard (i.e. ratio of base to crosslinker) and octane (as solvent) with three concentrations are developed. Each mixture is applied to the water filled membranes and allowed to be cured for 24 hrs at room temperature. Note that Octane evaporates during this period. That is, the water droplet are encapsulated with PDMS with different modulus. After, we performed FTIR analysis in transmission mode and the results are shown in **Supplementary Figure 18**. The results show that as the modulus of interface increases, due to the effect of surface factor on Gibbs energy barrier, ice nucleation temperature increases. This is another proof that the ice nucleation is heterogenous and not within the bulk of the liquid. This additional experiment also confirms that the soft octane-water interface leads to suppression of freezing to extremely low temperatures.

Supplementary Note 8. Effect of pores wall active sites on ice nucleation

We conducted ice formation experiments in pores just filled with water and no oil through FTIR metrology with different pore sizes. The results are shown in **Supplementary Figure 19**. As shown, ice nucleation temperature increases as the size decreases. In this case, the water has contact with the inner surface of the pores and the pore's walls act as nucleation sites.

Ice nucleation temperature as a function of pore dimension is shown in **Supplementary Figure 20**. Note that this is completely in contrast to the case that the oil-water interface exists in which ice nucleation temperature drops at lower pore dimensions.

The observed nucleation temperature could be explained through the thermodynamics of ice nucleation. In this case, nanodroplets experience negative pressure inside the pores due to the curvature of the water meniscus. Surface factor, $f(m,x)$, and chemical potential difference, $\Delta\mu$, are calculated for this scenario as a function of pore dimension and are plotted in **Supplementary Figure 21a**. As shown, as the size decreases, the surface factor decreases as well, primarily due to the concave interface of pore walls. In addition, the negative Laplace pressure in the nanodroplet decreases, which in turn leads to an increase in the chemical potential difference. The decrease in $f(m,x)$ and the increase in $\Delta\mu$ both lead to drops in the Gibbs energy barrier for ice nucleation, **Supplementary Figure 21b**.

We assume that oil wets the surface and water is encompassed by oil, as the oil has lower surface tension than water and the system is stable when the oil wets the pore walls. Thus, water does not have contact with pore walls and active sites. Also, even if we consider that some pores have defects or water has contact with the pore wall and freeze in higher temperatures, we can observe the changes in both electrical resistance and FTIR method, when most of the pores are frozen. In other words, freeing of few pores does not show significant changes in results and significant changes occur when the majority of pores are frozen.

To further demonstrate that pore walls (with their potential active nucleation sites) are not involved in ice nucleation, we calculated surface factor, $f(m,x)$, and chemical potential difference, $\Delta\mu$, for a *hypothetical* case where water contained inside the pores is surrounded by the oil at the ends, but oil does not wet the interior pore walls. Therefore, water is in direct contact with the walls within the pores. In this case, due to the curvature of the oil-water interface, there is a positive pressure build-up inside the water. The surface factor for the inner concave surface of pore walls is also known, thus Gibbs free energy can be obtained. As shown in **Supplementary Figure 22a**, upon decreasing the size, f and $\Delta\mu$ both decrease and the effect of f is more significant. Thus, ΔG^* must decrease (**Supplementary Figure 22b**) for smaller sizes which is in contrast with our experimental observations; hence this scenario fails i.e., the walls are wetted with the oil.

The concavity is defined with respect to viewing from inside the droplet. Due to the fact that ice nucleates inside the water not oil, the oil is a concave substrate for ice nucleation as shown in **Supplementary Figure 23**. Also, for a concave interface, ΔG^* decreases as the size of droplet decreases. (i.e. f(m,x) becomes smaller for smaller droplets).

Supplementary Note 9. Theoretical ice nucleation temperature of nanodroplets based on the classical nucleation theory (CNT)

Ice nucleation rate which is a function of Gibbs energy barrier is defined in Eq. (S9)[9,12].

$$R(T) = \frac{1}{\tau_{av}} = K\exp\left(-\frac{\Delta G^*}{k_B T}\right) \quad (S13)$$

Where τ_{av} is ice nucleation delay time and K is a kinetic constant and defined as follows[12]:

$$K = Z\beta N \quad (S10)$$

Where Z is Zeldovich non-equilibrium factor, β is the rate of addition of atoms or molecules to the critical nucleus and N is the number of atomic nucleation sites per unit volume. By substituting Eq. (S1) in Eq. (S9), we have

$$R = Kexp\left(-\frac{16\pi\gamma_{IW}^3 f(m)}{3k_B T(\rho\Delta\mu)^2}\right) \quad (S14)$$

Also, $\Delta\mu$ for a nanodroplet can be expressed as follows:

$$\Delta\mu_{Nd} = \Delta\mu_b + p\Delta v \quad (S15)$$

By substituting Eq. (S12) in Eq. (S11), we can write the ice nucleation rate of nanodroplets with respect to bulk water as[4]

$$\frac{R_{Nd}}{R_b} = \exp\left[-\frac{16\pi\,\gamma_{Iw}^3 f(m)}{3k_B T\rho^2}\left(\frac{1}{(\Delta\mu_b + p\Delta v)^2} - \frac{1}{\Delta\mu_b^2}\right)\right] \quad (S16)$$

$\Delta\mu_b$ can be estimated as[4]

$$\Delta\mu_b = \frac{\Delta H(T_m - T)}{T_m} \quad (S17)$$

Where $\Delta H = 271000 \frac{J}{kg}$ is the specific enthalpy of freezing[13]. By substituting Eqs. (S6) and (S14) into Eq. (S13), we get to

$$\alpha = T^{0.05}\left[\frac{1}{(998(T_m - T) + P\Delta v)^2} - \frac{1}{\left(998(T_m - T)\right)^2}\right] \quad (S18)$$

Where α is a constant and is written as

$$\alpha = -\frac{7.5*10^7*k_B*\rho^2*\ln\left(\frac{R_{Nd}}{R_b}\right)*f(m)}{16\pi} \quad (S19)$$

We calculated α by replacing Laplace pressure and the experimental T_N obtained for 150 nm nanodroplets in Eq. (S15). Then, this α was used to calculate T_N for the other sizes. Note that we considered at the nucleation temperature of each nanodroplet, the rate of nucleation is the same.

Furthermore, the ratio ($\frac{R_{Nd}}{R_b}$) is plotted as a function of size at T=-41°C in **Fig. 4d** in the main text and T=-10°C in **Supplementary Figure 24**.

Supplementary References

1. Liu, H. & Cao, G. Effectiveness of the Young-Laplace equation at nanoscale. *Sci. Rep.* **6**, 1–10 (2016).
2. Lo, Y. Relationships between the hydrophilic–lipophilic balance values of pharmaceutical excipients and their multidrug resistance modulating effect in Caco-2 cells and rat intestines. *J. Control. Release* **90**, 37–48 (2003).
3. Berry, J. D., Neeson, M. J., Dagastine, R. R., Chan, D. Y. C. & Tabor, R. F. Measurement of surface and interfacial tension using pendant drop tensiometry. *J. Colloid Interface Sci.* **454**, 226–237 (2015).
4. Li, T., Donadio, D. & Galli, G. Ice nucleation at the nanoscale probes no man's land of water. *Nat. Commun.* **4**, 1887 (2013).
5. Jaccard, C. Mechanism of the electrical conductivity in ice. *Ann. N. Y. Acad. Sci.* **125**, 390–400 (1965).
6. Felber, S. *Water Fundamentals Handbook*. (DRI-STEEM, 2017).
7. Irajizad, P., Nazifi, S. & Ghasemi, H. Icephobic surfaces: Definition and figures of merit. *Adv. Colloid Interface Sci.* **269**, 203–218 (2019).
8. Hakimian, A., Nazifi, S. & Ghasemi, H. Physics of Ice Nucleation and Growth on a Surface. *Ice Adhes.* 87–110 (2020) doi:10.1002/9781119640523.ch3.
9. Fletcher, N. H. Size Effect in Heterogeneous Nucleation. *J. Chem. Phys.* **29**, 572 (1958).
10. Eberle, P., Tiwari, M. K., Maitra, T. & Poulikakos, D. Rational nanostructuring of surfaces for extraordinary icephobicity. *Nanoscale* **6**, 4874–81 (2014).
11. Smith, M. L. *et al.* Force-Induced Unfolding of Fibronectin in the Extracellular Matrix of Living Cells. *PLoS Biol.* **5**, e268 (2007).
12. Russell, K. C. Nucleation in solids: The induction and steady state effects. *Adv. Colloid*

Interface Sci. **13**, 205–318 (1980).
13. Li, T., Donadio, D., Russo, G. & Galli, G. Homogeneous ice nucleation from supercooled water. *Phys. Chem. Chem. Phys.* **13**, 19807–19813 (2011).
